# Supplementary material for: Cachexia and Sarcopenia in Oligometastatic Non-Small Cell Lung Cancer: Making a Potential Curable Disease Incurable?
Source: Cancers (Basel). 2024 Jan 4;16(1):230. doi: 10.3390/cancers16010230 (PMC10777972; doi:10.3390/cancers16010230)
Supplement: Supplementary file 1 [file cancers-16-00230-s001.zip › Special issue Cancers - Table S1a.pdf]

Table S1a. Toxicities in patients with the intention of radical treatment stratified by cachexia and sarcopenia.

**Grading**	**A: No cachexia and no sarcopenia (n=133)**	**B: Cachexia, no sarcopenia (n=46)**	**C: Sarcopenia, no cachexia (n=34)**	**D: Cachexia and sarcopenia (n=21)**	**P value**
**1**	6	2	1	0	0.78
**2**	27	11	4	8	0.13
**3**	43	18	13	6	0.74
**4**	4	0	0	2	0.10
**5**	2	0	0	0	0.68
