# Supplementary material for: Cachexia and Sarcopenia in Oligometastatic Non-Small Cell Lung Cancer: Making a Potential Curable Disease Incurable?
Source: Cancers (Basel). 2024 Jan 4;16(1):230. doi: 10.3390/cancers16010230 (PMC10777972; doi:10.3390/cancers16010230)
Supplement: Supplementary file 1 [file cancers-16-00230-s001.zip › Special issue Cancers - Table S1b.pdf]

Table S1b. Toxicities in patients with sOMD NSCLC with the intention of radical treatment

**Toxicity**	**Grade 1**	**Grade 2**	**Grade 3**	**Grade 4**	**Grade 5**
**Alopecia**		1			
**Anemia**			18	2	
**Arterial injury**			1		
**Arterial thromboembolism**		1			
**Bone pain**			1		
**Bronchial obstruction**				1	
**Chest pain**			1	1	
**Colitis**		1			
**Chronic kidney disease**			4		
**Cystitis**	1	1			
**Delirium**			1		
**Diarrhea**	2	1	2		
**Duodenal ulcer**			1		
**Dysphasia**			1		
**Edema cerebral**					1
**Epistaxis**	1				
**Fatigue**	2	2	1		
**Febrile neutropenia**		2	10	1	1
**Fever**	1	2	4	1	
**Gastritis**			1		
**Gingival pain**			1		
**Guillan-Barre syndrome**				1	
**Hearing impaired**		1			
**Heart failure**			1		
**Hepatitis**		2	3		
**Herpes simplex reactivation**		1			
**Hypotension**			1		
**Hypothyroidism**		2			
**Laryngitis**		1			
**Lung infection**		2	3		
**Malaise**		1	6		
**Mucositis oral**		1			
**Nausea**	1	5	2		
**Peripheral motor neuropathy**		1			
**Peripheral sensory neuropathy**		1	1		
**Pleural effusion**		1			
**Pneumonitis**		1	1		
**Pneumothorax**		2	1		
**Radiation esophagitis**		3	4		
**Radiation pneumonitis**		5	3		
**Rash pustular**	1	4	1		
**Subcutaneous emphysema**			1		
**Thromboembolic event**		3	6		
